# Neurologisch-psychiatrische Begutachtung des Post-COVID-Syndroms

**DOI:** 10.1007/s00115-022-01292-4

**Published:** 2022-04-19

**Authors:** M. Tegenthoff, C. Drechsel-Schlund, B. Widder

**Affiliations:** 1 Neurologische Klinik und Poliklinik, BG-Universitätsklinikum Bergmannsheil, Ruhr-Universität Bochum, Bürkle-de-la-Camp Platz 1, 44789 Bochum, Deutschland; 2 Berufsgenossenschaft für Gesundheitsdienst und Wohlfahrtspflege, Hamburg, Deutschland; 3 Neurowissenschaftliche Gutachtenstelle, Bezirkskrankenhaus Günzburg, Günzburg, Deutschland

**Keywords:** Begutachtung, Fatigue, Post-COVID-Syndrom, Berufskrankheit, Arbeitsunfall, Expert opinion, Fatigue, Post-COVID syndrome, Occupational disease, Occupational accident

## Abstract

Infektionen mit dem „severe acute respiratory syndrome coronavirus 2“ (SARS-CoV-2) können zu einer Multiorganerkrankung und in der Folge zu sehr unterschiedlichen klinischen Manifestationen der „coronavirus disease 2019“ (COVID-19) führen. Neben akuten Symptomen kommt den Langzeitbeschwerden im Kontext der Infektion vermehrte Aufmerksamkeit zu, man spricht vom Long- oder Post-COVID-Syndrom. Mit Blick auf die Sozialversicherungssysteme werden gutachterliche Beurteilungen solcher Gesundheitsprobleme zunehmend an Bedeutung gewinnen, wobei neurologische und psychiatrische Symptome die häufigsten Beschwerden sind. Neben rechtlichen Grundlagen der medikolegalen Begutachtung mit Schwerpunkt auf der gesetzlichen Unfallversicherung werden in der vorliegenden Übersicht grundlegende Aspekte der Begutachtung diskutiert und Orientierungshilfen für die Begutachtung der häufigsten neurologischen und psychiatrischen Symptome des Post-COVID-Syndroms geboten.

**Zusatzmaterial online:**

Die Onlineversion dieses Beitrags (10.1007/s00115-022-01292-4) enthält weiterführende Literatur.

## Abstract (en)

Infections with severe acute respiratory syndrome coronavirus 2 (SARS-CoV-2) can lead to a multiorgan disease and subsequently to very different clinical manifestations of coronavirus disease 2019 (COVID-19). In addition to acute symptoms, the long-term complaints in the context of the infection, known as long or post-COVID syndrome, are increasingly attracting attention. With respect to social insurance systems, expert opinions of such problems will become more and more important, whereby neurological and psychiatric symptoms are the most frequent complaints. In addition to the legal principles of the medico-legal assessment with a focus on statutory accident insurance, this overview article discusses the principles of expert assessment and presents landmarks for the expert opinion of the most frequent neurological and psychiatric symptoms occurring in the context of post-COVID syndrome.

Das erstmals Ende 2019 beschriebene neue Coronavirus Typ 2 („severe acute respiratory syndrome coronavirus 2“, SARS-CoV-2) führte ab März 2020 zu der offiziell durch die World Health Organization (WHO) erklärten und bis heute anhaltenden COVID-19(„coronavirus disease 2019“)-Pandemie. Nachdem diese zunächst primär als schwere Atemwegsinfektion angesehen wurde, zeigte sich rasch, dass das neue Virus zu einer Multiorganerkrankung mit sehr vielgestaltigen klinischen Manifestationen und mit sehr unterschiedlichen Verläufen führen kann [1, 2]. Wie von anderen Virusinfektionen, z. B. mit dem Epstein-Barr-Virus, bekannt, finden sich zunehmend COVID-19-Erkrankte mit protrahierten, langdauernden klinischen Symptomen und subjektiven Beschwerden nach der akuten Infektionsphase.

## Definition und Häufigkeit

Eine nach einer SARS-CoV-2-Infektion auftretende, protrahierte Symptomatik wird bei einer Persistenz von mehr als 12 Wochen als Post-COVID-Syndrom bezeichnet. In Abgrenzung zur Akutphase der Infektion über eine Dauer von ca. 4 Wochen definiert die Leitlinie der Arbeitsgemeinschaft der Wissenschaftlichen Medizinischen Fachgesellschaften (AWMF) Post-COVID/Long-COVID [1] als ein postakutes prolongiertes symptomatisches Stadium zwischen der 4. und 12. Woche sowie eine „Long COVID Phase“, in der ab der 4. postinfektiösen Woche neue Symptome hinzutreten oder länger als 4 Wochen bestehen. Zwischen einem Post-COVID‑ und einem Long-COVID-Syndrom besteht insofern eher ein fließender Übergang. Im Alltag findet sich z. T. eine synonyme Begriffsverwendung. Im Folgenden wird für beide Phasen der Begriff Post-COVID-Syndrom (PCS) verwendet.

Ein PCS kann sowohl nach schweren als auch nach leichten klinischen Verläufen auftreten

Die Häufigkeit der PCS-Manifestationen schwankt in der bisherigen Literatur z. T. deutlich [27]. Dies ist wesentlich auf die heterogene Struktur der untersuchten Patientenpopulationen mit Unterschieden u. a. in Alter, Krankheitsschwere, Erhebungsmethodik oder Gruppengröße zurückzuführen [1]. Grundsätzlich kann ein PCS offensichtlich sowohl nach schweren als auch nach leichten klinischen Verläufen auftreten. Nach einem schweren Verlauf z. B. mit Langzeitbeatmung treten sicherlich nicht nur COVID-19-spezifische, sondern auch unspezifische Symptome auf, die wesentlich auf die intensivmedizinische Versorgung zurückzuführen sind.

## Gutachtlich relevante Symptome und Beschwerden

**Figure 1:**
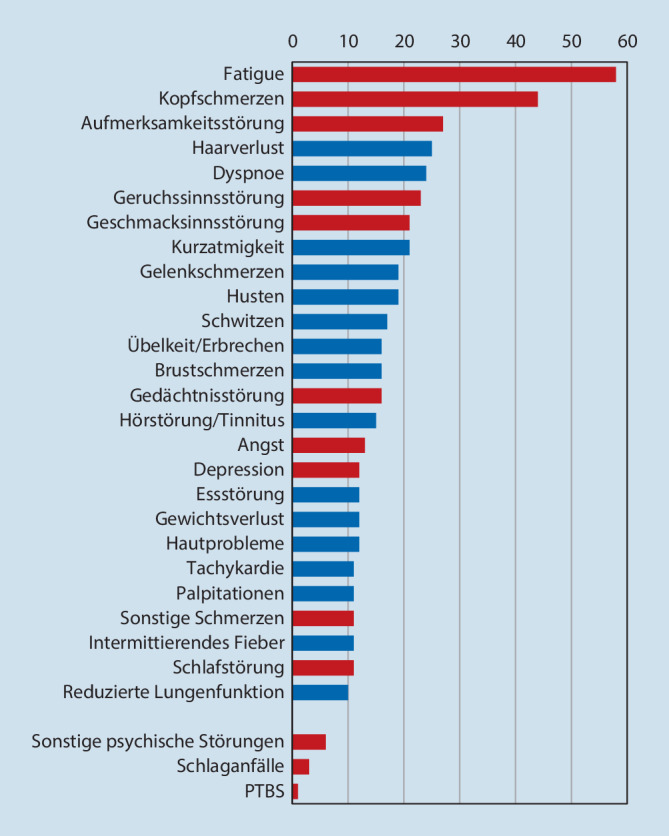

Betrachtet man die gesamte Gruppe der SARS-CoV-2-infizierten Patienten, so findet sich bei der überwiegenden Mehrzahl ein *milder unkomplizierter Akutverlauf* mit vollständiger Ausheilung ohne ein PCS. Soweit ein solches auftritt, ist die Bandbreite der subjektiv beklagten Beschwerden und der klinischen Symptome sehr groß (Abb. 1; [1, 6, 8, 20]).

Die keinesfalls vollständige Aufzählung der Beschwerden und Symptome macht deutlich, dass es sich beim PCS gutachtlich um eine fachübergreifende, interdisziplinäre Herausforderung handelt, bei der dem neurologisch-psychiatrischen Fachgebiet erhebliche Bedeutung zukommt. Eine wesentliche Problematik ergibt sich bei einer symptomorientierten fachspezifischen Begutachtung dadurch, dass trotz vielgestaltiger subjektiver Beschwerden die objektivierbare Organ- und Funktionsdiagnostik im neurologischen sowie u. a. auch pneumologischen und kardiologischen Bereich häufig unauffällig ist.

## Grundlegende Aspekte der Begutachtung

Selbst bei konservativer Schätzung der Häufigkeit des PCS resultiert allein aus der großen Zahl der COVID-19-Erkrankungen auch eine große Zahl von PCS-Betroffenen. Diese stammen überwiegend aus der berufstätigen Bevölkerung, sodass in naher Zukunft in verschiedenen Rechtsgebieten in zunehmender Häufigkeit gutachtliche Fragestellungen zu erwarten sind. In allen Rechtsgebieten ist dabei festzuhalten, dass dem Antragsteller von Versicherungsleistungen die objektive Beweislast für das Bestehen der geklagten Funktionsstörungen zukommt, ein „in dubio pro aegroto“ ist im rechtlichen Kontext nicht vorgesehen.

### Post-COVID-Syndrome in der gesetzlichen Unfallversicherung

Die Erkrankung infolge einer Infektion mit dem SARS-CoV-2-Virus kann ein Versicherungsfall in der gesetzlichen Unfallversicherung (GUV) sein und sowohl als Berufskrankheit oder als Arbeitsunfall anerkannt werden [7].

#### COVID-19-Erkrankung als Berufskrankheit.

Bei einer COVID-19-Erkrankung kommt eine Berufskrankheit „Infektionskrankheit“ nach § 9 Abs. 1 Sozialgesetzbuch (SGB) VII in Verbindung mit Nr. 3101 der Anlage 1 zur Berufskrankheitenverordnung (BK-Nr. 3101) dann in Betracht, wenn die betroffene Person zum einen im Gesundheitsdienst, in der Wohlfahrtspflege oder in einem Laboratorium tätig ist oder durch eine andere Tätigkeit der Infektionsgefahr „in erheblich höherem Grade als die übrige Bevölkerung ausgesetzt“ war und zum anderen bei ihr eine SARS-CoV-2-Infektion als sog. Gesundheits(erst)schaden vorgelegen hat. Sowohl die Infektionsquelle als auch die Infektion müssen im sog. „Vollbeweis“ nachgewiesen sein, die berufliche Verursachung muss zumindest überwiegend wahrscheinlich sein [7]. Diese Klärung obliegt in aller Regel dem Unfallversicherungsträger. Eine Ansteckung mit dem SARS-CoV-2-Virus durch private Kontakte im unversicherten Bereich kann der Anerkennung der COVID-19-Erkrankung als Berufskrankheit entgegenstehen.

#### COVID-19-Erkrankung als Arbeitsunfall.

Im Einzelfall kann eine COVID-19-Erkrankung auch einen Arbeitsunfall darstellen, wobei dieser definitionsgemäß den Nachweis eines Erregerkontakts („Unfallereignis“) während einer konkreten Arbeitsschicht voraussetzt.

Ist die SARS-CoV-2-Infektion als Gesundheits(erst)schaden bestätigt, ist auch die nachfolgende Symptomatik als Gesundheitsfolgeschaden im sog. „Vollbeweis“ nachzuweisen, für den Zusammenhang genügt im Bereich der GUV die hinreichende Wahrscheinlichkeit. Dies bedeutet, dass unter Berücksichtigung von Vorerkrankungen sowie anderer konkurrierender Ursachen den für den Zusammenhang sprechenden Umständen ein deutliches Übergewicht zukommt, sodass darauf die Überzeugung des Unfallversicherungsträgers oder des Sozialgerichts gestützt werden kann. Diese Klärung obliegt dem medizinischen Sachverständigen.

Entsprechend der höchstrichterlichen Rechtsprechung ist eine diagnostische Klassifikation des spezifischen Gesundheitsschadens nach den aktuellen Kodierungssystemen erforderlich. Für die Entscheidung über einen Entschädigungsanspruch reicht aber die Diagnosebezeichnung allein nicht aus. Vielmehr ist darüber hinaus notwendig, dass die aus dem Gesundheitsfolgeschaden resultierenden Funktionseinschränkungen nach Art und Ausmaß bzw. Schweregrad festgestellt werden [13].

### Gutachtliche Objektivierung

Bei der Klärung von Zusammenhangsfragen sind grundsätzlich zwei Formen der gutachtlichen Objektivierung zu unterscheiden:

- die direkte Objektivierung und
- die indirekte Objektivierung.

#### Direkte Objektivierung.

Ein derartiger Nachweis liegt vor, wenn der dokumentierte Verlauf einer Gesundheitsstörung zusammen mit bildgebenden und/oder messtechnischen Befunden (z. B. Elektrophysiologie, Labor) nach allgemeiner medizinischer Erfahrung klare Rückschlüsse auf bestehende Funktionsstörungen zulässt.

#### Indirekte Objektivierung.

Sind geklagte Beeinträchtigungen wie Schmerzen oder Fatigue-Symptome vorwiegend im subjektiven Erleben der zu Begutachtenden verhaftet, gilt es, möglichst viele Indizien zu sammeln, anhand derer dann eine „Brücke“ zwischen einem nachgewiesenen Schädigungsereignis und geltend gemachten Schädigungsfolgen gebildet werden kann (Tab. 1). Ergibt das gesamte Spektrum der Befunde ein in sich schlüssiges Bild, können die subjektiv geklagten Beeinträchtigungen dann im Sinne eines „Indizienbeweises“ in tatsächlich bestehende Funktionsstörungen „transferiert“ werden. Dieser Nachweis erfordert stets eine umfassende *Beschwerdenvalidierung* in Abgleich von Aktenlage, Exploration, Beobachtung und erhebbaren klinischen Befunden.

**Table 1:** 

Anknüpfungstatsache	Gutachtliches Kriterium
Erstsymptomatik	Nachweis einer infektionstypischen Erstsymptomatik unter Berücksichtigung des Schweregrades in geeignetem zeitlichem Zusammenhang zu dem Infektionsgeschehen anhand der Aktenlage in Korrelation zur Schilderung des Probanden im Rahmen der Begutachtung
Folgesymptomatik	Nachweis nachfolgend aufgetretener, charakteristischer Symptome der betreffenden Störung a) *Retrospektiv* anhand der Aktenlage in Korrelation zur Schilderung des Probanden im Rahmen der Begutachtung b) *Aktuell* anhand der im Rahmen der gutachtlichen Untersuchung erhobenen Befunde
Verlauf	Nachweis eines „nachvollziehbaren“ klinischen Verlaufs, auch in Bezug auf geeignete Rehabilitationsmaßnahmen
Konkurrierende Faktoren	Keine konkurrierenden Faktoren, die für die Symptomatik maßgeblich sind oder diese unterhalten
Konsistenz	Nachweis eines in sich schlüssigen Bildes in der Zusammenschau von Akten und klinischem Bild

^a^Je mehr positive Anknüpfungstatsachen vorliegen, umso sicherer ist die Objektivierung

## Begutachtung neurologisch-psychiatrisch relevanter Post-COVID-Syndrome

Bei der Begutachtung von Probanden mit geklagtem PCS sind zwei Situationen abzugrenzen:

### Mittelschwerer bis schwerer, z. T. lebensbedrohlicher Akutverlauf.

Relativ einfach erscheint die Beurteilung bei Infizierten mit einem mittelschweren bis schweren, z. T. lebensbedrohlichen Akutverlauf, oft auch mit der Notwendigkeit einer intensivmedizinischen Behandlung ggf. unter Einschluss einer extrakorporalen Membranoxygenierung (ECMO-Therapie). In diesen Fällen sind zumeist eindeutige *organpathologische Befunde* wie z. B. Lungengerüstveränderungen, Myokardläsionen, Thrombosen, Enzephalo‑/Myelitiden oder objektivierbare neuromuskuläre Störungen mit begleitenden typischen Veränderungen in der kardiopulmonalen und/oder neurologisch/neuropsychologischen Funktionsdiagnostik nachweisbar. In dieser Gruppe finden sich neben organischen Psychosyndromen z. B. im Rahmen einer Enzephalopathie (s. unten) auch psychoreaktive Störungen bis hin zur posttraumatischen Belastungsstörung.

### Primär asymptomatischer oder leicht- bis mittelschwerer Akutverlauf.

Schwierig zu erfassen und einzuordnen ist die vielgestaltige Symptomatik bei SARS-CoV-2-Infizierten mit einem primär asymptomatischen oder leicht- bis mittelschwerem Akutverlauf, die in der Regel ambulant, z. T. aber auch stationär behandelt wurden und die über einen langen Zeitraum von zum Teil mehr als einem Jahr nach der Infektion über persistierende unspezifische Beschwerdebilder klagen, *ohne dass eine korrespondierende Organpathologie* nachweisbar ist. Diese Beschwerdebilder treten z. T. nach einem „freien Intervall“ mit zunächst wiedererlangter Arbeitsfähigkeit erst Monate nach der Infektion auf. Im Vordergrund der geklagten Beschwerden steht zumeist eine persistierende körperliche und/oder kognitive Leistungsminderung im Sinne einer Fatigue, oft verbunden mit zahlreichen weiteren vielgestaltigen Beeinträchtigungen. Dazu gehören u. a. Wortfindungsstörungen, Parästhesien und Sensibilitätsstörungen, Koordinationsprobleme, Schluckstörungen, Muskel- und Gelenkschmerzen, Haarausfall, belastungsabhängige Dyspnoe, thorakales Beklemmungsgefühl, Tachykardien und verschiedenste psychische Beeinträchtigungen von Angst und Depression über Panikattacken bis hin zu dissoziativen Störungen. Diese Fallkonstellationen stellen insbesondere auch unter gutachtlichen Aspekten eine große Herausforderung dar, da die Kausalitätsbewertung schwierig und problematisch ist. Diese Problematik zeigt sich auch darin, dass die o. g. zumeist unspezifischen Beschwerden/Symptome auch von vermeintlichen COVID-19-Patienten geklagt werden, bei denen unter Berücksichtigung des serologischen Befundes gar keine SARS-CoV-2-Infektion abgelaufen ist [8, 22].

Sind relevante Funktionsstörungen nachgewiesen, ist im zweiten Schritt der Ursachenzusammenhang in Abgrenzung zu infektunabhängigen konkurrierenden Erkrankungen, Belastungsfaktoren und Aggravationstendenzen zu klären. Hierzu gehört auch die Heranziehung des Vorerkrankungsverzeichnisses. Im Einzelfall ist eine SARS-CoV-2-Infektion auch als Gelegenheitsursache im Rahmen einer davon unabhängigen z. B. schon länger dauernden Fehlentwicklung zu prüfen [14]. Die Bewertung von COVID-19-Erkrankungen orientiert sich für die gesetzliche Unfallversicherung bei der MdE(Minderung der Erwerbsfähigkeit)-Einschätzung an den vorhandenen Tabellenwerken (z. B. [29]), für das Versorgungs- und Schwerbehindertenrecht an den Versorgungsmedizinischen Grundsätzen.

### Fatigue-Symptomatik

Fatigue stellt in Studien das häufigste subjektive Symptom im Rahmen eines PCS dar [1, 2, 20]. Dieser Symptomkomplex aus rascher Ermüdbarkeit und physischer und/oder kognitiver Leistungsminderung ist seit langem im Gefolge anderer Viruserkrankungen oder im Zusammenhang mit Tumorerkrankungen oder z. B. der Multiplen Sklerose bekannt [29].

Im gutachtlichen Kontext ist zwischen „Fatigue“ und „Fatigability“ zu unterscheiden

Grundsätzlich begründet die Diagnose „Fatigue“ allein keine funktionelle Leistungseinschränkung. Im gutachtlichen Kontext empfiehlt sich eine Unterscheidung zwischen „Fatigue“ als subjektivem Gefühl einer vorzeitigen Ermüdung mit resultierender Leistungsminderung, welches anamnestisch oder mittels Fragebögen erfasst werden kann, und „Fatigability“ als einer objektiv mess- bzw. nachweisbaren Minderung der motorischen und/oder kognitiven Performance [18]. Dabei korreliert eine messbare Fatigability offensichtlich auch mit relevanten Funktionsbeeinträchtigungen [12].

Sofern das Bestehen einer Fatigability nicht durch objektivierbare organmedizinische (Funktions‑)Befunde im Sinne eines direkten Beweises nachweisbar ist, steht im Zentrum der gutachtlichen Bewertung eine Konsistenzprüfung. Hierzu gehört eine subtile Einzelfallanalyse der Alltagsaktivitäten sowie eine Evaluation möglicher Zielkonflikte mit sekundärem Krankheitsgewinn. Der Einsatz von Selbstbeurteilungsskalen und Fragebögen allein ist für eine Begutachtung unzureichend. Bei schwierigen Fragestellungen kommt ggf. eine mehrtägige Längsschnittbeobachtung durch geschultes medizinisches Personal z. B. im stationären Rahmen oder auch in Form einer standardisierten „Evaluation der funktionellen Leistungsfähigkeit“ (EFL) in Betracht.

Aber auch im Rahmen einmaliger gutachtlicher Untersuchungen können verschiedene Tests zur Beweisführung beitragen:

#### Kognitive Fatigability.

Eine kognitive Fatigability lässt sich durch eine differenzierte neuropsychologische Testdiagnostik unter Einschluss von Tests zur Daueraufmerksamkeit abklären, ggf. im zeitlichen Verlauf über den Tag oder nach Belastung. Dies erfordert in der Regel allerdings eine zeitintensive neuropsychologische Zusatzbegutachtung. Um intentionale Verzerrungen auszuschließen, gehören hierzu auch spezifische Verfahren der Beschwerdenvalidierung [30].

#### Motorische Fatigability.

Für die Erfassung der motorischen Fatigability existieren derzeit noch keine standardisierten bzw. konsentierten Erfassungsinstrumente. Diesbezügliche Ansätze verwenden in erster Linie repetitive Handkrafttests oder Veränderungen des Gangmusters bzw. der Ganggeschwindigkeit im Verlauf eines längeren körperlichen Belastungstests. Die Etablierung und Validierung standardisiert anwendbarer Messverfahren bleibt abzuwarten [11]. Im Vordergrund steht daher aktuell die Verhaltensbeobachtung und ggf. Konsistenzprüfung durch Einsatz klinischer Tests [29].

### Geruchs- und Geschmacksstörungen

Geruchs- und Geschmacksstörungen stellen ein häufiges Symptom bei der COVID-19-Erkrankung dar. Diese Symptomatik zeigt sich infolge einer direkten Schädigung des Riechepithels oder der Riechbahn insbesondere bei eher leichten Verlaufsformen sowie bei jungen, sonst weitgehend gesunden Patienten. Grundsätzlich können Geruchs- und Geschmacksstörungen Erstsymptom einer COVID-19-Infektion sein und im Verlauf auch als isolierte einzige klinische Symptomatik, zum Teil mit komplettem Verlust der sensorischen Funktion, bestehen [2]. Der Verlauf ist grundsätzlich günstig. In der Regel kommt es nach wenigen Wochen zu einer langsamen Rückbildung der Ausfallsymptomatik, die häufig mit Parosmien einhergehen kann. Nach einem Jahr ist in ca. 95 % der Fälle mit einer vollständigen Restitution der Geruchs- und Geschmacksfunktion zu rechnen.

Im gutachtlichen Kontext sollte die Erfassung von Geruchs- und Geschmacksstörungen nicht allein aufgrund der subjektiven Darstellung der Probanden, sondern durch den Einsatz standardisierter Riechtests (z. B. „sniffin’ sticks“) erfolgen [28], die Anwendung von Alternativwahlverfahren dient der Konsistenzprüfung. In unklaren Fällen ist auch eine Objektivierung mittels olfaktorisch und gustatorisch evozierter Potenziale möglich.

### Neurokognitive Störungen

Im gutachtlichen Kontext spielt eine differenzierte neuropsychologische Testdiagnostik unter Einschluss einer Beschwerdenvalidierung eine zentrale Rolle in der Objektivierung und Dokumentation der subjektiven Beschwerdesymptomatik (Tab. 1). Da testpsychologische Befunde ätiologisch unspezifisch sind und gutachtlich zusätzlich eine Abgrenzung nachweisbarer kognitiver Beeinträchtigungen zu infektunabhängigen Ursachen erfolgen muss, sind hier weitere Indizien (Anknüpfungstatsachen) von Bedeutung, die eine infektbedingte Schädigung bzw. Funktionseinschränkung des Gehirns belegen. So kann z. B. die Dokumentation eines schweren Akutverlaufs mit langdauerndem Delir eine Enzephalopathie anzeigen, die eine längerdauernde kognitive Störung begünstigen oder begründen kann. Die magnetresonanztomographische Bildgebung zeigt in der Regel keine infektspezifischen Veränderungen und ist insofern auch für die gutachtliche Sicherung einer zerebralen Schädigung wenig hilfreich.

Die kognitiven Beeinträchtigungen zeigen einen „Decrescendo-Charakter“

Mittels Positronenemissionstomographie (PET) ließ sich in der subakuten Phase der COVID-19-Erkrankung bei stationär behandlungsbedürftigen Patienten eine frontoparietal betonte Reduktion des zerebralen Glukosestoffwechsels nachweisen, die mit neuropsychologisch fassbaren Beeinträchtigungen korrelierte. In einer Verlaufsstudie ca. 6 Monate später fand sich eine Normalisierungstendenz der Stoffwechselminderung, die mit einer signifikanten, jedoch zu diesem Zeitpunkt noch unvollständigen Restitution der kognitiven Performance einherging [5, 15]. Diese Befunde sind nach aktuellem Kenntnisstand als Beleg für eine infektassoziierte zerebrale Funktionsstörung mit resultierenden Einschränkungen der Hirnleistungsfähigkeit einzuordnen. Wesentlich für die gutachtliche Bewertung ist dabei der offensichtliche „Decrescendo-Charakter“ der kognitiven Beeinträchtigungen, der ggf. durch Verlaufsuntersuchungen erfasst werden muss.

### Schmerzsyndrome

Im Rahmen eines PCS werden häufig unterschiedliche muskuloskelettal, myalgisch oder neuropathisch erscheinende oder auch unspezifische Schmerzsyndrome geklagt. Außer den ätiologisch bzw. organisch eindeutig zuordnungsfähigen Schmerzen z. B. im Rahmen einer „critical illness polyneuropathy“ (CIP) liegt bislang keine wissenschaftliche Evidenz bez. COVID-19-spezifischer Schmerzursachen vor [23]. Lang anhaltende Schmerzsyndrome sind gutachtlich daher in der Regel nur bei nachweisbarer Organpathologie als PCS anzuerkennen und erfordern eine strenge Konsistenzprüfung [3].

Wesentlich ist in diesem Zusammenhang auch die gutachtliche Bewertung der häufig von SARS-CoV-2-Infizierten längerfristig geklagten Kopfschmerzsymptomatik. Entsprechend den Erfahrungen mit anderen infektbedingten Kopfschmerzen erscheint eine vorübergehende infektbedingte Kopfschmerzsymptomatik auch über mehrere Monate z. B. im Rahmen einer länger dauernden generellen Leistungseinschränkung (s. unten) nachvollziehbar. Die Neuentstehung eines langanhaltenden Kopfschmerzsyndroms durch eine SARS-CoV-2-Infektion kann aktuell demgegenüber nicht als belegt angesehen werden. Die rein deskriptive Diagnose eines „new daily persistent headache“ allein begründet im gutachtlichen Kontext keine Kausalität [19].

### Psychische Gesundheitsstörungen

Die Wahrscheinlichkeit einer psychischen Gesundheitsstörung nach einer SARS-CoV-2-Infektion scheint gegenüber anderen Akuterkrankungen aus bisher nicht näher bekannten Gründen erhöht zu sein. Zwar finden sich erwartungsgemäß bei intensivmedizinisch behandelten COVID-19-Patienten infolge der lebensbedrohlichen Erkrankung gehäuft posttraumatische Belastungsstörungen [10]. Darüber hinaus besteht jedoch generell keine nachvollziehbare Korrelation zur Schwere der Akutphase. Aufgrund der inzwischen über zwei Jahre kontinuierlichen, ausgeprägten Medienpräsenz der COVID-19-Erkrankung muss bei der Bewertung möglicher psychischer PCS-Symptome daher insbesondere die Abgrenzung zu einer vermehrten Selbstbeobachtung/Selbstattribution bei primär ängstlichen Persönlichkeiten erfolgen [14].

Angststörungen und depressive Störungen werden am häufigsten berichtet

Am häufigsten wird im Rahmen eines PCS über Angststörungen und nachfolgend über depressive Störungen berichtet. Zusätzlich zeigen sich unspezifische Anpassungsstörungen, bei denen z. T. Schuldzuweisungen gegenüber dem Arbeitgeber bzw. Vorgesetzten z. B. aufgrund mangelnder Schutzmaßnahmen oder fehlender Wertschätzung von Bedeutung sind. Weiterhin kann es nach unserer Erfahrung zur Entwicklung dissoziativer Störungen bei zumeist iatrogener Fixierung nicht zuletzt aufgrund der aktuell noch unklaren pathophysiologischen Grundlagen des PCS kommen. Sofern diese mittelbare Folge einer schwerwiegenden COVID-19-Organerkrankung sind, ergeben sich gutachtlich selten Probleme. In anderen Fällen besteht bei krankheitswertigen Störungen aufgrund der multifaktoriellen Verursachung psychischer Erkrankungen im Allgemeinen hoher Begründungsbedarf, warum diese COVID-19-Folge sein sollen.

Wesentlich bei der gutachtlichen Beurteilung psychischer Störungen im möglichen Zusammenhang mit einer SARS-CoV-2-Infektion ist hinsichtlich der Kausalitätsbewertung die Kenntnis und Bewertung relevanter Vorerkrankungen sowie die Evaluation z. B. psychosozialer oder wirtschaftlicher Zielkonflikte, deren Bedeutung im Rahmen der Pandemiesituation eher an Bedeutung zugenommen hat. Die Begutachtung sollte unter Berücksichtigung der in der „Leitlinie zur Begutachtung psychischer und psychosomatischer Störungen“ dargestellten Grundlagen erfolgen [4].

### Meningoenzephalitiden/Myelitiden

Eher selten finden sich im Rahmen der SARS-CoV-2-Infektion Meningoenzephalitiden oder Myelitiden, wobei letztere zum Teil immunvermittelt mit einer zeitlichen Latenz auftreten können. In der Regel lassen sich diese Krankheitsbilder und ggf. deren Residuen aufgrund der typischen Organpathologien diagnostisch eindeutig objektivieren und hinsichtlich der resultierenden Funktionsstörungen gutachtlich zuordnen. Diese Infektionen des Zentralnervensystems sind unter einem klinischen Aspekt rein phänomenologisch nicht als erregerspezifisch anzusehen [24].

Häufiger zeigen sich im Verlauf der Akuterkrankung, aber auch als PCS, Enzephalopathien. Diesbezüglich sind angesichts der häufiger nach schweren Verläufen mit intensivmedizinischer Behandlungsnotwendigkeit auftretenden Symptomatik verschiedene Ursachen wie Hypoxie, septische Verläufe oder multiples Organversagen in Betracht zu ziehen. Da eine delirante Symptomatik in der Akutphase der Virusinfektion häufig ein klinisches Zeichen für eine Enzephalopathie und auch für eine schlechtere Prognose darstellt, bedarf es unter gutachtlichen Gesichtspunkten in diesen Fällen einer genauen Evaluation der initialen klinischen Verlaufsdokumentation [24].

### Schlaganfälle

Ischämische Hirninfarkte treten infektabhängig im Rahmen einer COVID 19-Infektion mit einer leicht erhöhten Häufigkeit insbesondere in den ersten Wochen nach der Infektion auf. Etwa 7‑fach seltener sind intrakranielle Blutungen. Patienten mit kardiovaskulären Risikofaktoren haben insofern ein erhöhtes Manifestationsrisiko. Es finden sich jedoch auch vereinzelt Schlaganfälle bei jüngeren Erkrankten mit einer Häufung großer Gefäßverschlüsse. Ursache ist am ehesten eine immunvermittelte Aktivierung des Gerinnungssystems oder das Auftreten vaskulärer Komplikationen im Rahmen anderweitiger Organbeteiligungen, z. B. bei Gefäßthrombosen [2, 25]. Im gutachtlichen Kontext ist hier eine Einzelfallanalyse mit kritischer Gewichtung relevanter vorbestehender Risikofaktoren erforderlich [29].

### Neuromuskuläre Erkrankungen

Eine neuromuskuläre Beteiligung kann bei COVID-19-Erkrankungen in unterschiedlicher Form auftreten. Im Rahmen der akuten Infektion oder mit einer geringen zeitlichen Latenz finden sich in eher geringer Zahl Erstmanifestationen einer Myositis in einem weiten klinischen Spektrum, welche neben einer entsprechenden klinischen Symptomatik mit Paresen – z. T. mit zeitlicher Latenz – eine Kreatinkinaseerhöhung sowie in der Regel magnetresonanztomographische Veränderungen in der betroffenen Muskulatur zeigen. Da eine immunologische Genese wahrscheinlich erscheint, ist die Gabe von Steroiden oder Immunglobulinen in der Regel erfolgreich. Für die gutachtliche Bewertung einer solchen z. T. auch längerfristig manifesten infektassoziierten myopathischen Symptomatik ist insofern eine genaue Kenntnis der initialen Befundkonstellation unverzichtbar [2, 26]. In sehr seltenen Fällen wird in Assoziation mit einer SARS-CoV-2-Infektion eine Verschlechterung vorbestehender neuromuskulärer Erkrankungen wie z. B. einer Myasthenia gravis oder einer chronisch inflammatorisch demyelinisierenden Polyneuropathie (CIDP) beschrieben [2].

Häufig wird über das Auftreten eines Guillain-Barré-Syndroms (GBS) in Assoziation mit einer COVID-19-Erkrankung berichtet. Dabei kann man wie bei anderen Viruserkrankungen am ehesten von einer unspezifischen postinfektiösen Genese ausgehen, wobei die Latenz zwischen der akuten SARS-CoV-2-Infektion und der Manifestation eines GBS mit oft 5 bis 10 Tagen relativ kurz zu sein scheint. Berichtet werden auch Fälle von GBS Wochen nach der akuten Infektion, wobei sich in diesen Fällen immer die Frage eines Kausalzusammenhangs stellt. Dies gilt insbesondere, da größere Studien auch keinen spezifischen Zusammenhang zwischen einer COVID-19-Erkrankung und einem GBS fanden, sodass die gutachtliche Beurteilung eines solchen Kausalzusammenhangs aktuell kontrovers zu diskutieren ist. Dabei ist gerade das Zeitintervall (s. oben) kritisch zu werten [16, 21]. Während die infektbedingte Auslösung von Polyneuritiden möglich erscheint, gibt es aktuell keine Hinweise auf die langfristige Entwicklung von Polyneuropathien in Assoziation mit einer SARS-CoV-2-Infektion. In der Akutphase sind Funktionsstörungen peripherer Nervenstrukturen feststellbar, die jedoch nach bisheriger Kenntnis nicht zu einer langdauernden peripheren Nervenerkrankung führen [2].

### Intensivmedizinische Komplikationen

Gerade im gutachtlichen Kontext wesentlich bedeutsamer sind aufgrund der größeren Häufigkeit „critical illness polyneuropathy“ (CIP) und „critical illness myopathy“ (CIM), welche typischerweise im Rahmen einer intensivmedizinischen Behandlung mit invasiver Beatmung auftreten können. Da die schweren Verläufe einer COVID-19-Erkrankung durch zum Teil wochen- bis monatelange intensivmedizinische Therapiephasen bestimmt sind, finden sich gehäuft Patienten, die langfristig unter entsprechenden z. T. hochgradigen funktionellen sensomotorischen Beeinträchtigungen leiden, was in der Regel kausal der Infektbehandlung zugeordnet werden kann [2, 9]. Davon in der Regel durch neurophysiologische Diagnostik abzugrenzen sind inaktivitätsbedingte Muskelatrophien, denen rein funktionell eine gleichartige Bedeutung zukommen kann.

Die z. T. lang dauernden, selbst noch nach mehr als einem Jahr geklagten Myalgien und/oder neuralgiformen Beschwerden bzw. Parästhesien sollten angesichts der aktuell noch begrenzten Kenntnis der Folgen einer SARS-CoV-2-Infektion grundsätzlich Anlass für eine umfassende neuromuskuläre Diagnostik sein, um manifeste Organschäden als Grundlage für die Beschwerden nicht zu übersehen. Ergänzend sollte in diesen Fällen auch an psychoreaktive Folgen gedacht und dies ggf. psychiatrisch/psychologisch abgeklärt werden.

## Zusammenfassung

Die Begutachtung des PCS stellt angesichts des komplexen, noch nicht vollständig verstandenen Krankheitsbildes nach einer SARS-CoV-2-Infektion eine interdisziplinäre Herausforderung dar. Neben der fachspezifischen Beurteilung infektbedingter Organschäden liegt die wesentliche Problematik in der gutachtlichen Objektivierung und Kausalitätsbewertung der vielgestaltigen subjektiven Beschwerdebilder, insbesondere bei einer geklagten Fatigue-Symptomatik. Grundvoraussetzung für jede gutachtliche Beurteilung in der gesetzlichen Unfallversicherung ist eine gesicherte SARS-CoV-2-Infektion (in der Regel mittels PCR-Virus- oder Antikörpernachweis), im zweiten Schritt dann eine abgeschlossene symptomorientierte Organdiagnostik zur Sicherung bzw. zum weitestgehenden Ausschluss infektbedingter Organschäden [17]. Eine vollständige Dokumentation des Krankheitsverlaufs und ein Vorerkrankungsverzeichnis sind dabei für die Bewertung unabdingbar.

Die Diagnose PCS muss im sog. Vollbeweis gestellt werden

Im gutachtlichen Kontext muss auch beim PCS die Diagnose im sog. Vollbeweis gestellt werden und sich die Kausalitätsbeurteilung auf den aktuellen wissenschaftlichen Kenntnisstand stützen. Auch wenn die Folgen einer SARS-CoV-2-Infektion noch nicht vollständig verstanden sind, haben sich grundlegende pathophysiologische Erkenntnisse/Abläufe nicht geändert. Insofern können z. B. einzelne Fallberichte allein nicht zur Grundlage einer Kausalitätsbeurteilung gemacht werden. Da die klinische Beschwerdesymptomatik auch der COVID-19-Erkrankung in der überwiegenden Mehrzahl der Fälle einen Decrescendo-Verlauf zeigt, muss bei einer anderen Verlaufsdynamik diese im gutachtlichen Kontext diskutiert und begründet werden. Da Erfahrungen hinsichtlich des PCS-Langzeitverlaufs bislang nicht existieren, sollte bei Feststellung einer manifesten Funktionsstörung eine Nachbegutachtung nach etwa ein (bis zwei) Jahren routinemäßig empfohlen werden.

Der wissenschaftliche Erkenntnisgewinn bezüglich der SARS-CoV-2-Infektion und des PCS ist aktuell angesichts von z. T. mehr als 1000 neuen Publikationen zu dieser Thematik pro Woche enorm. Gesicherte Studienergebnisse können insofern den aktuellen wissenschaftlichen Kenntnisstand zukünftig verändern. Dies kann eine Anpassung der dargestellten Eckpunkte einer gutachterlichen Bewertung des PCS erforderlich machen.

## Fazit für die Praxis

- Neurologisch-psychiatrische Fragestellungen stellen im Rahmen der interdisziplinären Begutachtung des Post-COVID-Syndroms (PCS) einen wesentlichen Anteil dar.
- Für den Bereich der gesetzlichen Unfallversicherung kann – je nach Voraussetzung – eine COVID-19(„coronavirus disease 2019“)-Erkrankung sowohl als Berufskrankheit als auch als Arbeitsunfall anerkannt werden.
- Eine SARS-CoV-2(„severe acute respiratory syndrome coronavirus 2“)-Infektion als Gesundheits(erst)schaden und das Vorliegen einer manifesten Funktionsstörung als Gesundheitsfolgeschaden im Rahmen eines PCS müssen im Vollbeweis gesichert werden.
- Eine gutachtlich wesentliche Problematik bei nachweisbaren Funktionsstörungen besteht in der kausalen Abgrenzung zu infektunabhängigen Erkrankungen sowie zu psychischen/lebensgeschichtlichen Einflussfaktoren.
- Bei der gutachtlichen Bewertung einer Fatigue-Symptomatik ist, sofern keine erklärenden organpathologischen Befunde vorliegen, stets eine eingehende Beschwerdenvalidierung – ggf. mit umfassender neuropsychologischer und körperlicher Leistungsdiagnostik – zur Konsistenzprüfung der geklagten Beschwerden erforderlich.

## Supplementary Information





## References

[1] AWMF-Leitlinie (Reg. Nr. 020-027): Post-Covid/Long-Covid. https://www.awmf.org/leitlinien/detail/ll/020-027.html. Zugegriffen: 21. Febr. 2022

[2] AWMF-Leitlinie (Reg.Nr. 030-144) Neurologische Manifestationen bei COVID-19. https://www.awmf.org/uploads/tx_szleitlinien/030-144l_S1_Neurologische_Manifestationen_bei_COVID-19_2021-12.pdf (Erstellt: 20. Dez. 2021). Zugegriffen: 21. Febr. 2022

[3] AWMF-Leitlinie (Reg.Nr. 094-003): Ärztliche Begutachtung von Menschen mit chronischen Schmerzen. https://www.awmf.org/leitlinien/detail/ll/094-003.html. Zugegriffen: 21. Febr. 2022

[4] AWMF-Leitlinie (Reg.Nr. 051-029): Begutachtung psychischer und psychosomatischer Störungen. https://www.awmf.org/leitlinien/detail/ll/051-029.html. Zugegriffen: 21. Febr. 2022

[5] Blazhenets G, Schroeter N, Bormann T, Thurow J, Wagner D, Frings L, Weiller C, Meyer PT, Dressing A, Hosp JA (2021). Slow but evident recovery from neocortical dysfunction and cognitive impairment in a series of chronic COVID-19 patients. J Nucl Med.

[6] Blomberg B, Mohn KG, Brokstad KA, Zhou F, Linchausen DW, Hansen BA, Lartey S, Onyango TB, Kuwelker K, Sævik M, Bartsch H, Tøndel C, Kittang BR, Cox RJ, Langeland N N, Bergen COVID-19 Research Group (2021). Long COVID in a prospective cohort of home-isolated patients. Nat Med.

[7] Brandenburg St, Woltjen M (2021). COVID-19 als Berufskrankheit oder Arbeitsunfall. MedSach.

[8] Bungenberg J, Humkamp K, Hohenfeld C, Rust MI, Ermis U, Dreher M, Hartmann NK, Marx G, Binkofski F, Finke C, Schulz JB, Costa AS, Reetz K (2022). Long COVID-19: Objectifying most self-reported neurological symptoms. Ann Clin Tranl Neurol.

[9] Cabañes-Martínez L, Villadóniga M, González-Rodríguez L, Araque L, Díaz-Cid A, Ruz-Caracuel I, Pian H, Sánchez-Alonso S, Fanjul S, Del Álamo M, Regidor I (2020). Neuromuscular involvement in COVID-19 critically ill patients. Clin Neurophysiol.

[10] Deng J, Zhou F, Hou W, Silver Z, Wong CY, Chang O, Huang E, Zuo QK (2021). The prevalence of depression, anxiety, and sleep disturbances in COVID-19 patients: a meta-analysis. Ann N Y Acad Sci.

[11] Dettmers C, Broscheid KC, Peters A, Hoogerbeets O, Sailer S, Wolff W, Vieten M, Jögbes M, Penner IK (2021). Motorische Fatigue und Fatigability bei MS. Fatigue bei Multipler Sklerose.

[12] Dettmers C, Marchione S, Weimer-Jaekel A, Godde B, Joebges M (2021). Cognitive Fatigability, not Fatigue predicts employment status in patients with MS three months after rehabilitation. Mult Scler Relat Disord.

[13] Drechsel-Schlund C (2020). Funktionseinschränkungen bei psychischen Unfallfolgen und MdE-Bewertung – aus Sicht der gesetzlichen Unfallversicherung. Med Sach.

[14] Dreßing H, Meyer-Lindenberg A (2021). „Long-Covid“ und „Post-Covid“ in der psychiatrischen Begutachtung. Med Sach.

[15] Hosp JA, Dressing A, Blazhenets G, Bormann T, Rau A, Schwabenland M, Thurow J, Wagner D, Waller C, Niesen WD, Frings L, Urbach H, Prinz M, Weiller C, Schroeter N, Meyer PT (2021). Cognitive impairment and altered cerebral glucose metabolism in the subacute stage of COVID-19. Brain.

[16] Keddie S, Pakpoor J, Mousele C, Pipis M, Machado PM, Foster M, Record CJ, Keh RYS, Fehmi J, Paterson RW, Bharambe V, Clayton LM, Allen C, Price O, Wall J, Kiss-Csenki A, Rathnasabapathi DP, Geraldes R, Yermakova T, King-Robson J, Zosmer M, Rajakulendran S, Sumaria S, Farmer SF, Nortley R, Marshall CR, Newman EJ, Nirmalananthan N, Kumar G, Pinto AA, Holt J, Lavin TM, Brennan KM, Zandi MS, Jayaseelan DL, Pritchard J, Hadden RDM, Manji H, Willison HJ, Rinaldi S, Carr AS, Lunn MP (2021). Epidemiological and cohort study finds no association between COVID-19 and Guillain-Barre syndrome. Brain.

[17] Kersten J, Baumhardt M, Hartveg P, Hoyo L, Hüll E, Imhof A, Kropf-Sanchen C, Nita N, Mörike J, Rattka M, Andreß S, Scharnbeck D, Schmidtke-Schrezenmeier G, Tadic M, Wolf A, Rottbauer W, Buckert D (2021). Long COVID: distinction between organ damage and deconditioning. J Clin Med.

[18] Kluger BM, Krupp LB, Enoka RM (2013). Fatigue and fatigability in neurologic illnesses: proposal for a unified taxonomy. Neurology.

[19] Lobo R, Wang M, Lobo S, Bahra A (2021). Time to retire ‘new daily persistent headache’: mode of onset of chronic migraine and tension-type headache. Cephalalgia.

[20] Lopez-Leon S, Wegman-Ostrosky T, Perelman C, Sepulveda R, Rebolledo PA, Cuapio A, Villapol S (2021). More than 50 long-term effects of COVID-19: a systematic review and meta-analysis. Sci Rep.

[21] Luijten LWG, Leonhard SE, van der Eijk AA, Doets AY, Appeltshauser L, Arends S, Attarian S, Benedetti L, Briani C, Casasnovas C, Castellani F, Dardiotis E, Echaniz-Laguna A, Garssen MPJ, Harbo T, Huizinga R, Humm AM, Jellema K, van der Kooi AJ, Kuitwaard K, Kuntzer T, Kusunoki S, Lascano AM, Martinez-Hernandez E, Rinaldi S, Samijn JPA, Scheidegger O, Tsouni P, Vicino A, Visser LH, Walgaard C, Wang Y, Wirtz PW, Ripellino P, Jacobs BC, IGOS consortium (2021). Guillain-Barré syndrome after SARS-CoV-2 infection in an international prospective cohort study. Brain.

[22] Matta J, Wiernik E, Robineau O, Carrat F, Touvier M, Severi G, de Lamballerie X, Blanché H, Deleuze JF, Gouraud C, Hoertel N, Ranque B, Goldberg M, Zins M, Lemogne C, Pratiques S, Relations et Inégalités Sociales en Population Générale Pendant la Crise COVID-19–Sérologie (SAPRIS-SERO) Study Group (2022). Association of self reported COVID-19 infection and SARS-CoV-2 serology test results with persistent physical symptomsamong french adults during the COVID-19 pandemic. JAMA Intern Med.

[23] Meyer-Frießem CH, Gierthmühlen J, Baron R, Sommer C, Üçeyler N, Enax-Krumova EK (2021). Pain during and after COVID-19 in Germany and worldwide: a narrative review of current knowledge. Pain Rep.

[24] Misra S, Kolappa K, Prasad M, Radhakrishnan D, Thakur KT, Solomon T, Michael BD, Winkler AS, Beghi E, Guekht A, Pardo CA, Wood GK, Hsiang-Yi Chou S, Fink EL, Schmutzhard E, Kheradmand A, Hoo FK, Kumar A, Das A, Srivastava AK, Agarwal A, Dua T, Prasad K (2021). Frequency of neurologic manifestations in COVID-19: a systematic review and meta-analysis. Neurology.

[25] Nannoni S, de Groot R, Bell S, Markus HS (2020). Stroke in COVID-19. A systematic review and meta-analysis. Int J Stroke.

[26] Saud A, Naveen R, Aggarwal R, Gupta L (2021). COVID-19 and myositis: what we know so far. Curr Rheumatol Rep.

[27] Sudre CH, Murray B, Varsavsky T, Graham MS, Penfold RS, Bowyer RC, Pujol JC, Klaser K, Antonelli M, Canas LS, Molteni E, Modat M, Cardoso JM, May A, Ganesh S, Davies R, Nguyen LH, Drew DA, Astley CM, Joshi AD, Merino J, Tsereteli N, Fall T, Gomez MF, Duncan EL, Menni C, Williams FMK, Franks PW, Chan AT, Wolf J, Ourselin S, Spector T, Steves CJ (2021). Attributes and predictors of long COVID. Nat Med.

[28] Whitcroft KL, Hummel T (2019). Clinical diagnosis and current management strategies for olfactory dysfunction: a review. JAMA Otolaryngol Head Neck Surg.

[29] Widder B, Gaidzik P (2018). Neurowissenschaftliche Begutachtung.

[30] Widder B, Penner IK (2021). Begutachtung und sozialmedizinische Beurteilung. Fatigue bei Multipler Sklerose.

