# Supplementary material for: Neurological and psychiatric expert assessment of post-COVID syndrome
Source: Nervenarzt. 2022 Apr 19;93(8):804–11. [Article in German] doi: 10.1007/s00115-022-01292-4 (PMC9017073; doi:10.1007/s00115-022-01292-4)
Supplement: Supplementary file 1 [file 115_2022_1292_MOESM1_ESM.docx]

# Weiterführende Literatur zum Beitrag

# Neurologisch-psychiatrische Begutachtung des Post-Covid-Syndroms

# M. Tegenthoff (1), C. Drechsel-Schlund (2), B. Widder (3)

# (1) Neurologische Klinik und Poliklinik, BG-Universitätsklinikum Bergmannsheil, Ruhr-Universität Bochum

# (2) Berufsgenossenschaft für Gesundheitsdienst und Wohlfahrtspflege, Hamburg

# (3) Neurowissenschaftliche Gutachtenstelle am Bezirkskrankenhaus Günzburg

**Weiterführende Literatur:**

1. AWMF-Covid-19 Leitlinien: https://www.awmf.org/die-awmf/awmf-aktuell/aktuelle-leitlinien-und-informationen-zu-covid-19/covid-19-leitlinien.html. Zugegriffen: 21.02.2022

# 2. AWMF-Leitlinie (Reg.Nr. 017-050): Riech- und Schmeckstörungen. https://www.awmf.org/leitlinien/detail/ll/017-050.html. Zugegriffen: 21.02.2022

# 3. AWMF-Leitlinie (Reg.Nr. 094-001): Allgemeine Grundlagen der medizinischen Begutachtung. https://www.awmf.org/leitlinien/detail/ll/094-001.html. Zugegriffen: 21.02.2022

4. Aladawi M, Elfil M, Abu-Esheh B, Abu Jazar D, Armouti A, Bayoumi A, Piccione E (2021) Guillain Barre Syndrome as a Complication of COVID-19: A Systematic Review. Can J Neurol Sci 5:1-11

5. Alwan NA (2021) The road to addressing Long Covid. Science 373:491-493

6. Augustin M, Schommers P, Stecher M, Dewald F, Gieselmann L, Gruell H, Horn C, Vanshylla K, Cristanziano VD, Osebold L, Roventa M, Riaz T, Tschernoster N, Altmueller J, Rose L, Salomon S, Priesner V, Luers JC, Albus C, Rosenkranz S, Gathof B, Fätkenheuer G, Hallek M, Klein F, Suárez I, Lehmann C. (2021) Post-COVID syndroime in non-hosipalised patients with COVID-19: a longitunal prospective cohort study. Lancet regional health 6:100122

7. Bitirgen G, Korkmaz C, Zamani A, Ozkagnici A, Zengin N, Ponirakis G, Malik RA. (2021) Corneal confocal microscopy identifies corneal nerve fibre loss and increased dendritic cells in patients with long COVID. Br J Ophthalmol 26: 319450.

8. Boesl F, Audebert H, Endres M, Prüss H, Franke C. (2021) A Neurological Outpatient Clinic for Patients With Post-COVID-19 Syndrome - A Report on the Clinical Presentations of the First 100 Patients. Front Neurol 12:738405.

9. Chaudhuri A, Behan PO. (2004) Fatigue in neurological disorders. Lancet 363:978-88.

10. Evans RW, Turner DP. (2021) Clinical features of new daily persistent headache: A retrospective chart review of 328 cases. Headache 61:1529-1538

11. Finsterer J, Scorza FA, Fiorini AC (2021) SARS-CoV-2-associated Guillain-Barre syndrome in 62 patients. Eur J Neurol 28:e10-e12

12. Groff D, Sun A, Ssentongo AE, Ba DM, Parsons N, Poudel GR, Lekoubou A, Oh JS, Ericson JE, Ssentongo P, Chinchilli VM (2021) Short-term an long-term rates of postacute sequelae of SARS-CoV-2 infection. JAMA Network Open 4:e2128568

13. Gudziol H, Guntinas-Lichius O (2019): Electrophysiologic assessment of olfactory and gustatory function. Handb Clin Neurol 164:247-262

14. Gupta L, Lilleker JB, Agarwal V, Chinoy H, Aggarwal R (2021) COVID-19 and myositis - unique challenges for patients. Rheumatology 60:907-910

15. Helms J, Kremer S, Merdji H, Schenck M, Severac F, Clere-Jehl R, Studer A, Radosavljevic M, Kummerlen C, Monnier A, Boulay C, Fafi-Kremer S, Castelain V, Ohana M, Anheim M, Schneider F, Meziani F. (2020) Delirium and encephalopathy in severe COVID-19: a cohort analysis of ICU patients. Crit Care 24:491.

16. Hotopf M, Bullmore E, O'Connor RC, Holmes EA. (2020) The scope of mental health research during the COVID-19 pandemic and its aftermath. Br J Psychiatry 217:540-542

17. Huang C, Huang L, Wang Y, Li X, Ren L, Gu X, Kang L, Guo L, Liu M, Zhou X, Luo J, Huang Z, Tu S, Zhao Y, Chen L, Xu D, Li Y, Li C, Peng L, Li Y, Xie W, Cui D, Shang L, Fan G, Xu J, Wang G, Wang Y, Zhong J, Wang C, Wang J, Zhang D, Cao B. (2021) 6-month consequences of COVID-19 in patients discharged from hospital: a cohort study. Lancet 397:220-232.

18. Jafri MR, Zaheer A, Fatima S, Saleem T, Sohail A. (2022) Mental health status of COVID-19 survivors: a cross sectional study. Virol J 19:3.

19. Marshall M. (2021) The four most urgent questions about long COVID. Nature 594:168-170.

20. Marx P, Widder B. (2018) Hirngefäßerkrankungen. In : Widder B, Gaidzik PW (Hrsg.) Neurowissenschaftliche Begutachtung, ed 3., Thieme, Stuttgart, pp 505–515.

21. Merten T. (2020) Beschwerdenvalidierung in der psychologischen und medizinischen Begutachtung. Prax Rechtspsychologie 30:59–76.

22. Munz M, Wessendorf S, Koretsis G, Tewald F, Baegi R, Krämer S, Geissler M, Reinhard M. (2020) Acute transverse myelitis after COVID-19 pneumonia. J Neurol 267:2196-2197.

23. Nalbandian A, Sehgal K, Gupta A, Madhavan MV, McGroder C, Stevens JS, Cook JR, Nordvig AS, Shalev D, Sehrawat TS, Ahluwalia N, Bikdeli B, Dietz D, Der-Nigoghossian C, Liyanage-Don N, Rosner GF, Bernstein EJ, Mohan S, Beckley AA, Seres DS, Choueiri TK, Uriel N, Ausiello JC, Accili D, Freedberg DE, Baldwin M, Schwartz A, Brodie D, Garcia CK, Elkind MSV, Connors JM, Bilezikian JP, Landry DW, Wan EY. (2021) Post-acute COVID19-syndrome. Nat Med 27:601-615

24. Renaud M, Thibault C, Le Normand F, Mcdonald EG, Gallix B, Debry C, Venkatasamy A. (2021) Clinical Outcomes for Patients With Anosmia 1 Year After COVID-19 Diagnosis. JAMA Netw Open 4:e2115352.

25. Research NIoH. Living with COVID-19 – Second review - a dynamic review of the

evidence around ongoing Covid19 (often called Long Covid) (2021) https://evidence.nihr.ac.uk/themedreview/living-with-covid19-second-review/. Zugegriffen 21.02.2022

26. Sehle A, Vieten M, Sailer S, Mündermann A, Dettmers C. (2014): Objective assessment of motor fatigue in multiple sclerosis: the Fatigue index Kliniken Schmieder (FKS). J Neurol 261:1752-62

27. Shao SC, Lai CC, Chen YH, Chen YC, Hung MJ, Liao SC. (2021) Prevalence, incidence and mortality of delirium in patients with COVID-19: a systematic review and meta-analysis. Age Ageing. 50:1445-1453.

28. Smith, B. (1995) The Russian Influenza in the United Kingdom, 1889-1894. Soc Hist Med 8:55-73

29. Ulmer, M. (2020) Bewertung und Verwertbarkeit neurologisch-psychiatrischer Gutachten. MedSach 116:134-139

30. Widder B. (2017) Klinische Tests zur Beschwerdenvalidierung. Fortschr Neurol Psychiatr 85:740–746.

31. Zou LQ, Linden L, Cuevas M, Metasch ML, Welge-Lüssen A, Hähner A, Hummel T. (2019) Self-reported mini olfactory questionnaire (Self-MOQ): A simple and useful measurement for the screening of olfactory dysfunction. Laryngoscope 130:E786-E790
